# Temperature and pH growth preferences predict the pathogenicity and environmental distributions of nontuberculous mycobacteria

**DOI:** 10.1128/msystems.00211-26

**Published:** 2026-07-21

**Authors:** Matthew J. Gebert, Nabeeh A. Hasan, Madeline R. Pill-Kastens, Lady Grant, Michael Hoffert, L. Elaine Epperson, Michael Strong, Noah Fierer

**Affiliations:** 1 Department of Ecology and Evolutionary Biology, University of Colorado 1877 , Boulder, Colorado, USA; 2 Cooperative Institute for Research in Environmental Sciences, University of Colorado 1877 , Boulder, Colorado, USA; 3 Center for Genes, Environment, and Health, National Jewish Health 2930, Denver, Colorado, USA; University of Memphis, Memphis, Tennessee, USA

**Keywords:** mycobacteria, nontuberculous mycobacteria, NTM, pathogenicity

## Abstract

The genus *Mycobacterium* is highly diverse, and in addition to *Mycobacterium tuberculosis*, it includes many other pathogenic and non-pathogenic taxa which are collectively referred to as the nontuberculous mycobacteria. Although a subset of nontuberculous mycobacteria are of known clinical significance, the specific traits that dictate pathogenicity remain largely undetermined. We hypothesized that pathogenic nontuberculous mycobacteria are likely to have optimal growth at low pH (~pH 4.5–5.5) and at temperatures near that of the human body (~37°C), enabling survival within human tissues. To test this hypothesis, we measured the growth preferences of 20 strains of nontuberculous mycobacteria, spanning the breadth of diversity across the genus, using cellular activity as a proxy for growth *in vitro*. Pathogens tended to share preferences for optimal growth at lower pH and higher temperature conditions, while those of limited or no clinical significance generally had growth optima outside a range that would be conducive to effective growth within the human body. We then used our *in vitro* measurements of pH preferences to determine if we could predict the environmental distributions of nontuberculous mycobacteria, as determined from a cultivation-independent survey of 143 soils across the United States. We show that the measured *in vitro* pH preferences effectively predicted the distributions of nontuberculous mycobacteria in soil. Our work highlights the broad range in pH and temperature preferences across the genus, demonstrates the utility of these measurements for differentiating human pathogens from non-pathogens, and describes how measured growth preferences can be used to predict the environmental distributions of nontuberculous mycobacteria.

**IMPORTANCE:**

The genus *Mycobacterium* includes *Mycobacterium tuberculosis* as well as over 200 taxa collectively referred to as the nontuberculous mycobacteria (NTM). Although most nontuberculous mycobacteria have limited clinical significance, a handful of species within the genus are responsible for a growing number of chronic infections worldwide—yet the traits that underlie their pathogenicity remain largely undetermined. We measured the growth preferences of 20 strains of nontuberculous mycobacteria *in vitro*, finding that NTM pathogens tended to share preferences for optimal growth at lower pH and higher temperature (similar to conditions encountered in a human host), while those of no clinical significance generally had growth optima outside the conditions of the human body. We then used our *in vitro* measurements to show that we could predict the distributions of nontuberculous mycobacteria in soil, an important environmental reservoir and potential source of exposure and subsequent infection. Our work highlights not only the broad range in pH and temperature preferences across the genus, but the utility of measuring these traits for understanding NTM pathogenicity and ultimately, advancing our understanding of the ecology of nontuberculous mycobacteria.

## INTRODUCTION

The bacterial genus *Mycobacterium* includes the pathogens that cause tuberculosis (*Mycobacterium tuberculosis*) and leprosy (*Mycobacterium leprae*). In addition to these two well-known pathogens, the genus also includes a broad range of other human and animal pathogens, as well as non-pathogenic environmental strains, that are collectively referred to as nontuberculous mycobacteria (NTM) (1, 2). The degree of pathogenicity varies greatly across the genus, with some NTM frequently implicated in opportunistic infections of humans (including the *Mycobacterium avium* complex, *Mycobacterium abscessus*, *Mycobacterium fortuitum*, and *Mycobacterium chelonae*). These infections present most commonly as pulmonary infections in patients with underlying lung disease or susceptibilities (3–8). NTM infections are of growing concern worldwide (7, 9) and often require extensive and costly treatments (10, 11). However, the majority of NTM taxa are non-pathogenic to humans, or are at least rarely associated with human disease (12). It remains unclear what specific traits differentiate pathogenic from non-pathogenic NTMs.

Pathogenic NTM that are implicated in human disease are distributed across the diversity of the genus. It has remained difficult to identify genes or specific traits that are shared by pathogenic members of this group (13–15). Rather, pathogenicity in humans likely stems from the capacity for certain NTM species to survive and proliferate within host tissue, using various intrinsic cellular strategies to evade the immune response, resulting in chronic inflammation, tissue deterioration and damage, and subsequent disease (16). Additionally, few genomic attributes or measurable traits have been identified that consistently, and reliably, differentiate pathogenic and non-pathogenic NTM strains (17).

We hypothesize that a key feature distinguishing pathogenic from non-pathogenic NTM is their ability to grow well at temperatures close to the human body temperature, allowing for survival and proliferation within human tissues. More specifically, we hypothesize that those NTM that commonly cause pulmonary infections should have higher temperature optima (~37°C) than non-pathogenic NTM or those NTMs that typically infect human skin, where temperatures are lower than human core body temperatures. This expectation arises from observations that *M. marinum* and *M. ulcerans* preferentially infect the extremities of the human body, which tend to be ~33°C–37°C (18), and propagate within hosts with cooler core body temperatures (19, 20). Likewise, we hypothesized that pathogenic NTM should grow better at more acidic pH values (4.5 to 6) than non-pathogenic NTM. This expectation is based on the observation that pathogenic NTMs can survive and potentially proliferate within the phagosome and phagolysosome of macrophages, presumably because they are able to withstand the acidic environment (typically ~pH 4.5–5.5) of phagocytic vacuoles (21–23). Although there is anecdotal evidence to suggest that the pH and temperature preferences of mycobacteria can be highly variable across members of the genus (24–29), reliable data on growth responses across gradients in temperature and/or pH are unavailable for nearly all NTM species. In summary, we hypothesized that the measured pH and temperature preferences for growth would be associated with the capability of NTM species to successfully infect humans.

One important feature of NTM infections is that they are environmentally acquired (30, 31). In contrast to *M. tuberculosis* and *M. leprae*, which are transmitted primarily human-to-human or zoonotically, NTM infections are primarily acquired from environmental sources. Such environmental sources may include soils (29, 32), residential plumbing (33–36), and freshwater lakes or streams (3, 37, 38). However, even within a given environment, NTM are not necessarily ubiquitous. The specific NTM taxa found within a given source environment can vary as a function of environmental conditions, as highlighted in recent biogeographical studies of showerhead biofilms (33) and surface soils (29), environments that are frequently implicated as potential sources of NTM infections in humans. While NTM were found to be common in these environments, there was a high degree of variation in the NTM taxa detected across samples from these environments, with some samples harboring NTM of clinical importance and others dominated by non-pathogenic NTM (29, 33). Understanding the biogeographical distributions of NTM in source environments is crucial for identifying the most likely sources of NTM infections and predicting when exposures to source environments are most likely to be of concern. Such knowledge of sources and habitats could be leveraged to minimize or mitigate potential infectious environmental exposures. In addition, characterizing NTM distributions in potential source environments is key to understanding the observed geographic variation in NTM disease prevalence across regions (39, 40). Currently, we have a limited ability to predict *a priori* what types of environments will harbor potentially pathogenic NTM capable of causing disease in humans.

While many factors likely influence what types of NTM are found where, we expect that NTM distributions in environmental samples are strongly influenced by pH and temperature conditions. This expectation follows from work in soil and freshwater systems highlighting that the abundances and types of NTM found in source environments can often vary as a function of pH and temperature (32, 41–44). Thus, we expect that information on the pH and temperature growth preferences of NTM will enable us to infer which environments are most likely to harbor specific NTM taxa, including NTM species of clinical significance. Instead of solely documenting biogeographical patterns in NTM distributions, we ultimately aim to be able to infer their distributions from information on their environmental preferences.

Here, we used laboratory-based assays to assess the growth responses of 20 unique NTM strains, isolated from a wide range of environments, across gradients in pH and temperature. Our goal was to quantify the variation in growth preferences across the genus *Mycobacterium*, testing the hypothesis that pathogenic NTM would have growth optima closer to human host conditions (acidic pH, higher temperatures) than non-pathogenic NTM. In addition, we assessed whether we could predict the environmental distributions of specific mycobacterial strains (including known pathogens) by considering their growth preferences *in vitro*. We did so by comparing the measured *in vitro* growth responses across pH gradients to the observed distributions of those NTM across 143 soils, demonstrating the feasibility of using *in vitro* assays of growth preferences to predict NTM distributions in source environments. Ultimately, this work expands our trait-based understanding of the genus *Mycobacterium* and highlights the importance of considering variation in environmental preferences for understanding the ecology and clinical significance of NTM.

## MATERIALS AND METHODS

### Mycobacterial isolates

NTM isolates used in this study were obtained from soil, premise plumbing biofilms, and from the American Type Culture Collection (ATCC, Manassas, VA, USA) and the German Collection of Microorganisms and Cell Cultures GmbH (DSMZ, Braunschweig, Germany), see Table S1. NTM cultures from premise plumbing were isolated from biofilm swabs using a standard NTM culturing protocol (33). NTM from soil were isolated by vortexing 1 to 5 g of soil in 10 mL of sterile 1× PBS/Tween 80 (final concentration: 0.02%) in 50-mL conical tubes to dislodge cells from soil particles. The soil mixture was then spun down at 500 × *g* for 3 min to allow larger debris to settle. To capture a broad diversity of mycobacteria from the environment, we chose not to use traditional decontamination methods, such as cetylpyridinium chloride, on the soil mixture prior to plating (45, 46). We then spread 1–25 µL of the soil supernatant on 10-cm diameter petri plates containing solid Middlebrook 7H11 without the addition of the oleic acid, albumin, dextrose, catalase supplement (OADC). Plates were incubated at both 25°C or 33°C for up to 12 weeks to capture slower-growing NTM. Individual colonies were then picked into 2-mL 96-well plates containing 500 µL of Middlebrook 7H9 without the addition of albumin, dextrose, and catalase (ADC), and incubated at 25°C for up to 4 weeks. Wells were then screened using mycobacterial-specific heat shock protein (*hsp65*) primers (47), and positive wells were streaked to purity on solid Middlebrook 7H10 and Middlebrook 7H11, and Sanger sequenced using the same *hsp65* primers to re-confirm strain identity.

**Fig 1:**
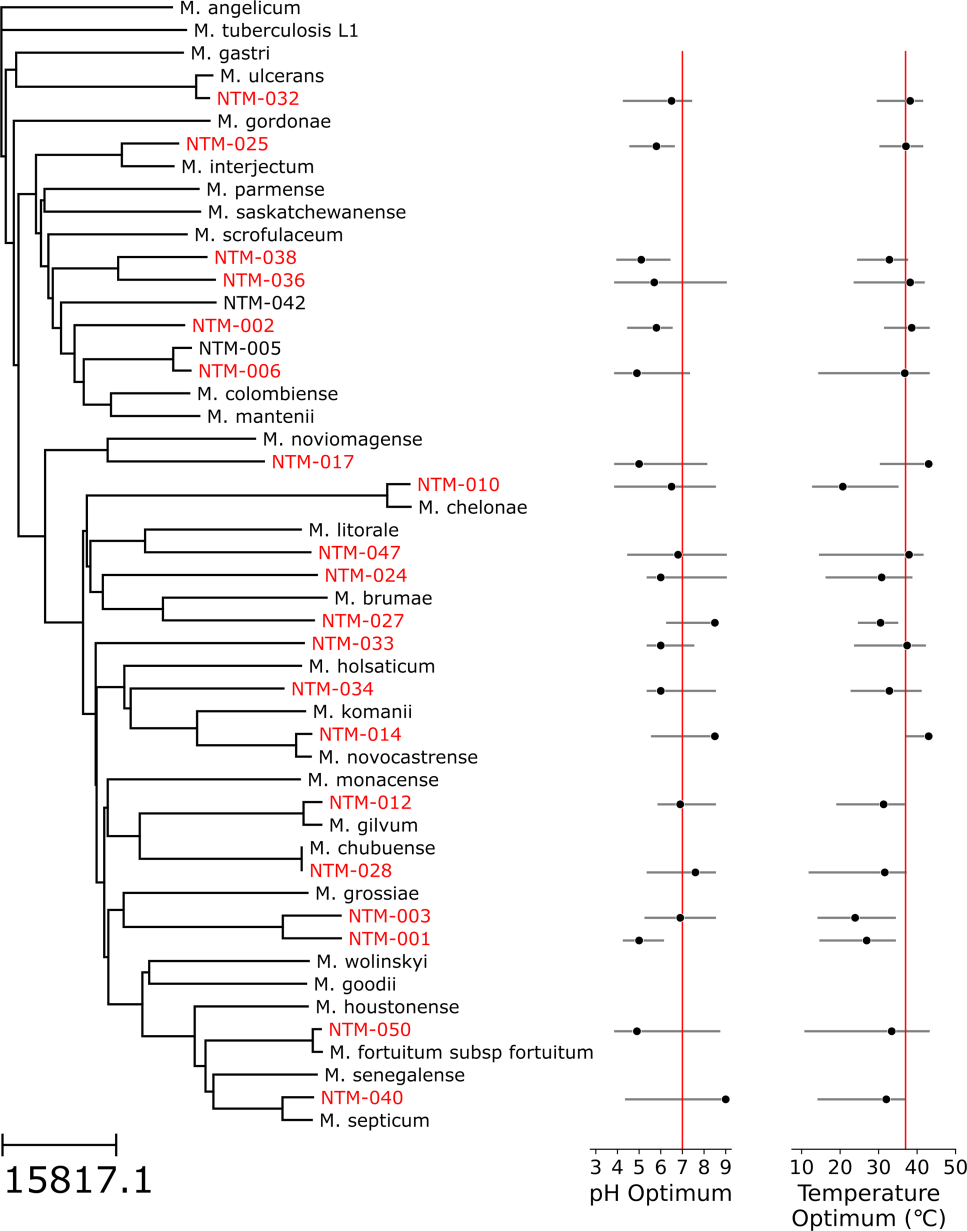
Phylogenetic relatedness, pH, and temperature optima of mycobacteria. Maximum likelihood phylogeny was based on 887 concatenated core genes shared between the 20 experimental (red) isolates and 26 reference NTM isolates (black) from GTDB. The measured pH and temperature (°C) optima for the experimental isolates included in this study are visualized, as well as the optimal range measured for each isolate (conditions where growth was within 50% of optimum; see Materials and Methods). The vertical red line in each plot indicates the traditional culturing conditions used for growth of mycobacteria. Additional marker gene trees showing the phylogenetic placement of the isolates within the genus are included in Fig. S1.

We included 20 total mycobacterial isolates that spanned the known diversity of the genus (Fig. 1), including nine that are deemed clinically significant in humans and 11 that do not routinely cause human disease. We chose to consider *Mycobacterium* as a single genus, rather than by the proposed four additional genera (48), to maintain consistency with the clinical literature (49, 50). Since clinical significance within the genus has been primarily assessed on a case-by-case basis, and pathogen designations can be subjective or based on a limited number of clinical cases, isolates in this experiment were considered “pathogenic” to humans (Table S1) if they are listed in the Official ATS/IDSA Statement (8, 51) as being the cause of clinical disease. A summary of all isolates included in this study, the source of each isolate, inferred taxonomy (see below), and likely pathogenicity is provided in Table S1.

### Phylogenetic and genomic analyses of the NTM isolates

DNA for whole genome sequencing (WGS) was extracted using the DNeasy UltraClean Microbial Kit (Qiagen, Germantown, MD, USA) according to the manufacturer’s instructions from swabs taken from the purity streak of the same isolate used to inoculate starter cultures for the assay experiments (described below). DNA was then stored at 4°C in elution buffer until sequencing. Aliquots of genomic DNA were used to prepare the libraries for sequencing as described previously (52), using the Illumina DNA Prep kit, (M) Tagmentation (P/N 20060059), with sequencing conducted on an Illumina MiSeq running the 2 × 300 bp sequencing chemistry, aiming for approximately 40× genomic read depth coverage on average.

Genomes were assembled with Unicycler (version 0.5.0) (53) and annotated with Prokka (version 1.14.5) (54), with pan-genomic analyses performed with Panaroo (version 1.2.6) (55). Sequence alignments were created from 887 concatenated core genes, and phylogenetic trees were generated with Mega X11 (56), as described previously (57), and using the ETE3 Toolkit (58), with the resulting trees (Fig. 1; Fig. S1), including both the 20 isolates included in this study, plus 26 additional reference NTM isolates representing the nearest named genome representative in the Genome Taxonomy Database (https://gtdb.ecogenomic.org/). The taxonomic identity of the novel mycobacterial isolates used in the experiment was determined based on proximity to known database isolates in the Genome Taxonomy Database (GTDB Release 08-RS214).

### Assays to quantify pH and temperature preferences

Assays were conducted in pre-sterilized 96-well deep-well culture plates (Axygen, Corning Life Sciences, Corning, NY, USA) with seven 96-well plates per isolate (one plate for each of the seven temperatures). Middlebrook 7H9 medium without ADC was added to each of the seven columns on each plate, with each column (eight replicate wells per column) adjusted to each of the six different pH levels (pH 4, 5, 6, 7, 8, and 9), with column 7 used as an uninoculated control (not pH adjusted, pH ~6.8). We prepared seven identical bottles of media in acid-washed 1-L glass bottles (Fisher Scientific, Waltham, MA, USA) to fill the assay plates, with pH adjusted using 12.1 N HCl and 1 N NaOH, and measured at room temperature (~22°C) using an Orion Star A211 pH meter. The medium bottles were then autoclaved for 30 min at 121°C. Medium pH was then re-measured at room temperature, post-autoclaving, and final pH values were recorded. Medium pH stability at 4°C was checked at room temperature after 4 months to ensure no appreciable changes occurred during storage. Prior to inoculating the plates and performing the assays, each plate was sealed with sterile sealing mats (Axygen, Corning Life Sciences, Corning, NY, USA) and stored at 4°C in the dark until use.

To prepare inocula for the pH and temperature growth assays, colonies were picked from solid media and grown in 7 mL of Middlebrook 7H9 agar base with the addition of Middlebrook ADC growth supplement (Sigma Aldrich, St. Louis, MO, USA) at 30°C in a shaking incubator (140 rpm) until turbidity was visible. One exception was *Mycobacterium xenopi*, which was grown at 38°C under static conditions, due to its poor growth at lower incubation temperatures. For a given strain, we inoculated six columns of each plate with a standardized inoculum concentration, with an additional column left uninoculated to serve as background controls for the ATP assays (columns 8–12 were empty). Inoculum concentrations were determined via ATP luminescence (see below), and 1 µL of inoculum at ~20,000 RLU was added to each well of the assay plate.

Plates were incubated at seven different temperatures to collect representative growth data for the prediction model—11°C, 22°C, 24°C, 30°C, 36°C, 38°C, and 43°C—selected to capture the range of temperatures NTM may be exposed to in the environment and in the human body. Mycobacterial growth in each well was inferred via ATP luminescence assays (BacTiter-Glo Microbial Cell Viability Assay, following manufacturer’s instructions) as more traditional methods for measuring cell growth in liquid culture, namely optical density, are less effective for mycobacteria as cells often clump together due to the hydrophobicity of their cell wall (59), resulting in inaccurate turbidity readings (13, 60). Instead, we consider ATP bioluminescence, which measures cellular activity, as a reliable proxy for mycobacterial growth (61–63). ATP luminescence readings were taken on day 0 to ensure that all initial inoculations resulted in ATP levels that were at, or below, detection, followed by one subsequent reading on day 7, and day 14 for each isolate. Then, 50 μL of culture from each well was used for each time point, mixed at a 1:1 ratio with the ATP luminescence buffer. As a few strains (for example, *Mycobacterium xenopi*) exhibited a high degree of clumping by day 14 with corresponding heterogeneity in ATP measurements, pH and temperature preferences were determined from the day 7 time points. At each of these three time points (days 0, 7, and 14), we conducted 392 individual ATP assays per isolate—eight replicate wells per column and six inoculated columns per plate (for the six different pH levels) and one uninoculated column per plate to serve as a background control, with one plate for each of the seven different temperatures.

### Determining the optimum growth conditions for each mycobacterial strain

To quantify the growth responses of each strain across the gradients in pH and temperature, we used the “mgcv” package (version 1.8-41) (64) in the R software package to fit a generalized additive model (GAM) to the measured luminescence values (code available at https://github.com/gebertm/Myco-Growth-Optima). The highest and lowest of the eight replicate readings per isolate at a given pH and temperature were removed, resulting in six replicate luminescence readings per pH and temperature for each isolate. Because of the potential interactions between pH and temperature in determining growth responses, each term was not considered separately in the model. The model was fit using the restricted maximum likelihood method (REML), with family = gaussian, link function = identity, and six basis functions (*k* = 6). All assumptions were checked for each model using gam.check in mgcv (output for gam.check from each individual model is included in Table S2). We then used the predict function to predict the luminescence values for all potential combinations of pH and temperature over our given experimental range. This allowed us to determine the pH and temperature combination that corresponded to the highest luminescence reading, which we consider the optimal growth condition for each isolate. Additionally, we defined the optimal growth range as being all pH and temperature conditions within 50% of the highest luminescence value (growth optima) as determined by the model. The final full predicted luminescence readings across the ranges in pH and temperature were visualized using the contour plot function in Plotly for R (65) to visualize both the optima and range in pH and temperature growth responses.

### Relating NTM distributions in soil to measured pH preferences

We next sought to determine whether the measured pH preferences of NTM strains (as determined from the *in vitro* growth assays described above) were informative for understanding NTM distributions in the environment, focusing on the soil environment given that NTM are nearly ubiquitous in soil (3, 66–68) and exposures to soil are often cited as a source of NTM infections (66, 69). As soil temperatures at individual locations can exhibit a high degree of variability (70), we focused on pH, testing the hypothesis that NTM strains are more likely to be found in those soils with pH values close to their measured pH optimum for growth, as determined from the *in vitro* assays. To test this hypothesis, we used a cultivation-independent data set that characterized NTM diversity via marker gene sequencing of the *hsp65* gene in 143 distinct soils collected from across the United States representing a broad range in soil characteristics, including measured soil pH values ranging from 4 to 9 (29). We matched the *hsp65* gene sequences from each of our screened isolates to the representative *hsp65* sequences from the (29) data set, where > 500 distinct amplicon sequence variants (ASVs) were detected across all 143 soils. We set a match threshold of 96% similarity in BLAST (71), identifying five isolates from our assays that matched four corresponding soil ASVs with <16 bp difference in the sequenced portion of the *hsp65* gene, with two of the five isolates matching to one single ASV within the *M. avium* complex (*M. avium* and *M. intracellulare*). This resulted in *M. avium* and *M. intracellulare* detected in 12 soils, *M. interjectum* detected in 8 soils, and *M. chubuense* and *M. novocastrense* detected in 5 and 41 soils, respectively.

### Statistical analysis

All statistical analyses were conducted in the R software package (R version 4.4.2, R Core Team 2024), and Figs. 3 and 4 were made using the ggplot package in R (72). To test whether NTM known to be clinically significant in humans were statistically different from those with little to no clinical significance, we ran a logistic regression model using the glm function (family = binomial) and a χ test between the residual deviance and the null deviance. Isolates were considered either clinically significant (1) or not (0) in the model based on the categorization in (8). The data were confirmed to meet the assumptions of the model. To test whether there was a correlation between our *in vitro* pH optima measurements and the distribution of matched mycobacterial taxa in soil, we ran a Spearman Rank correlation (cor.test, method = spearman).

## RESULTS

### Large variation in pH and temperature optima across the NTM isolates

We focused on 20 mycobacterial isolates, spanning the breadth of diversity across the genus *Mycobacterium* (Fig. 1), with these isolates derived from soil and residential plumbing biofilms from a variety of geographic locations (Table S1). Representative isolates that were difficult or rarely isolated from the environment (*M. interjectum*) or filled important phylogenetic gaps in our collection (*M. marinum*, *M. xenopi*) were purchased from culture collections (see Materials and Methods and Table S1). Additionally, we focused on NTM that range in their degree of pathogenicity to humans, from frequently opportunistic taxa (e.g., *M. chelonae, M. fortuitum,* and members of the *M. avium* complex) to taxa that are rarely, or never, reported as pathogenic (e.g., *M. hodleri* and *M. gilvum*).

**Fig 2:**
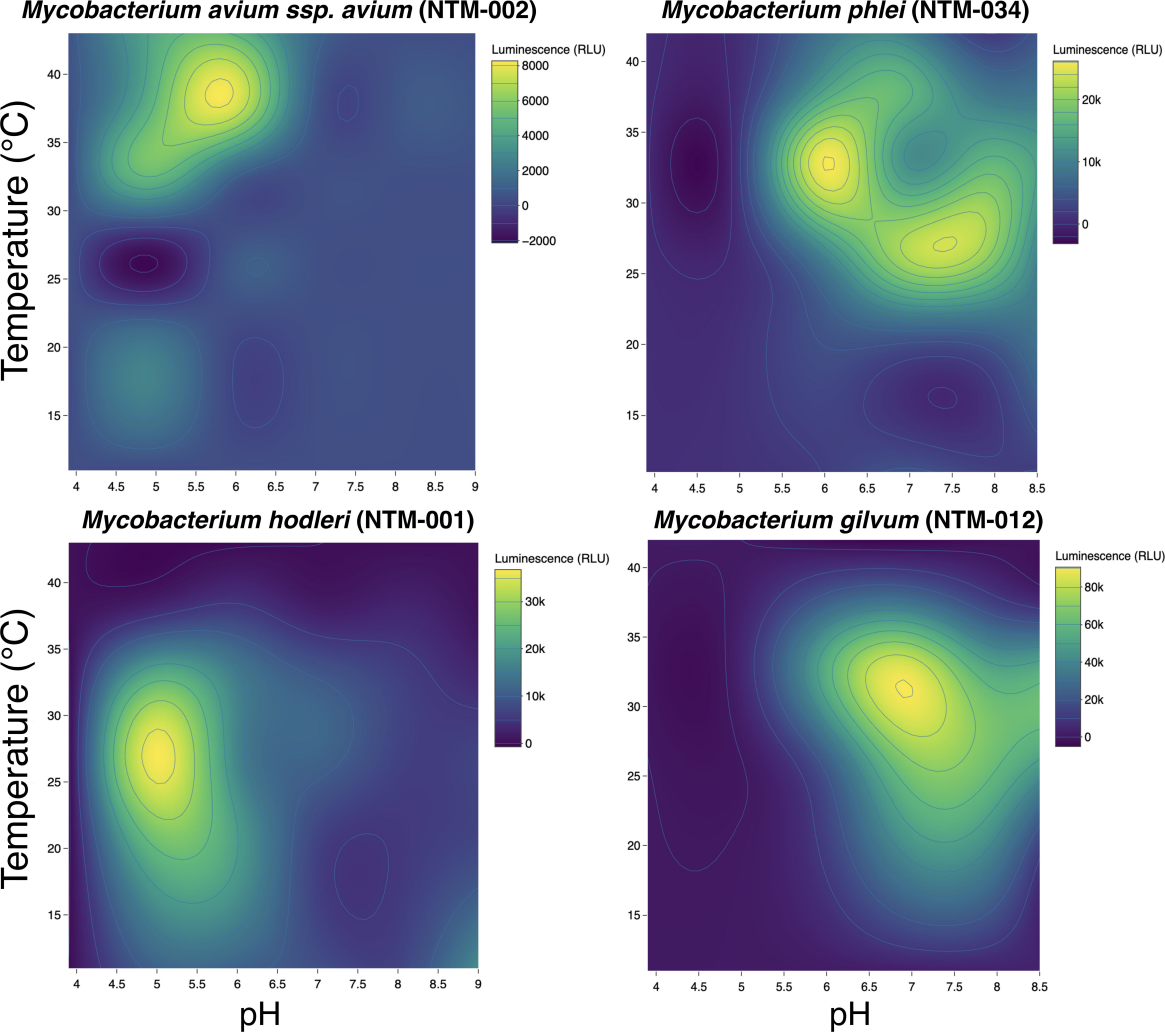
Examples of variation in the pH and temperature preferences of four NTM isolates. Contour plots showing ATP bioluminescence readings (each taken on Day 14). See Fig. S2 for contour plots from all isolates included in this study.

**Fig 3:**
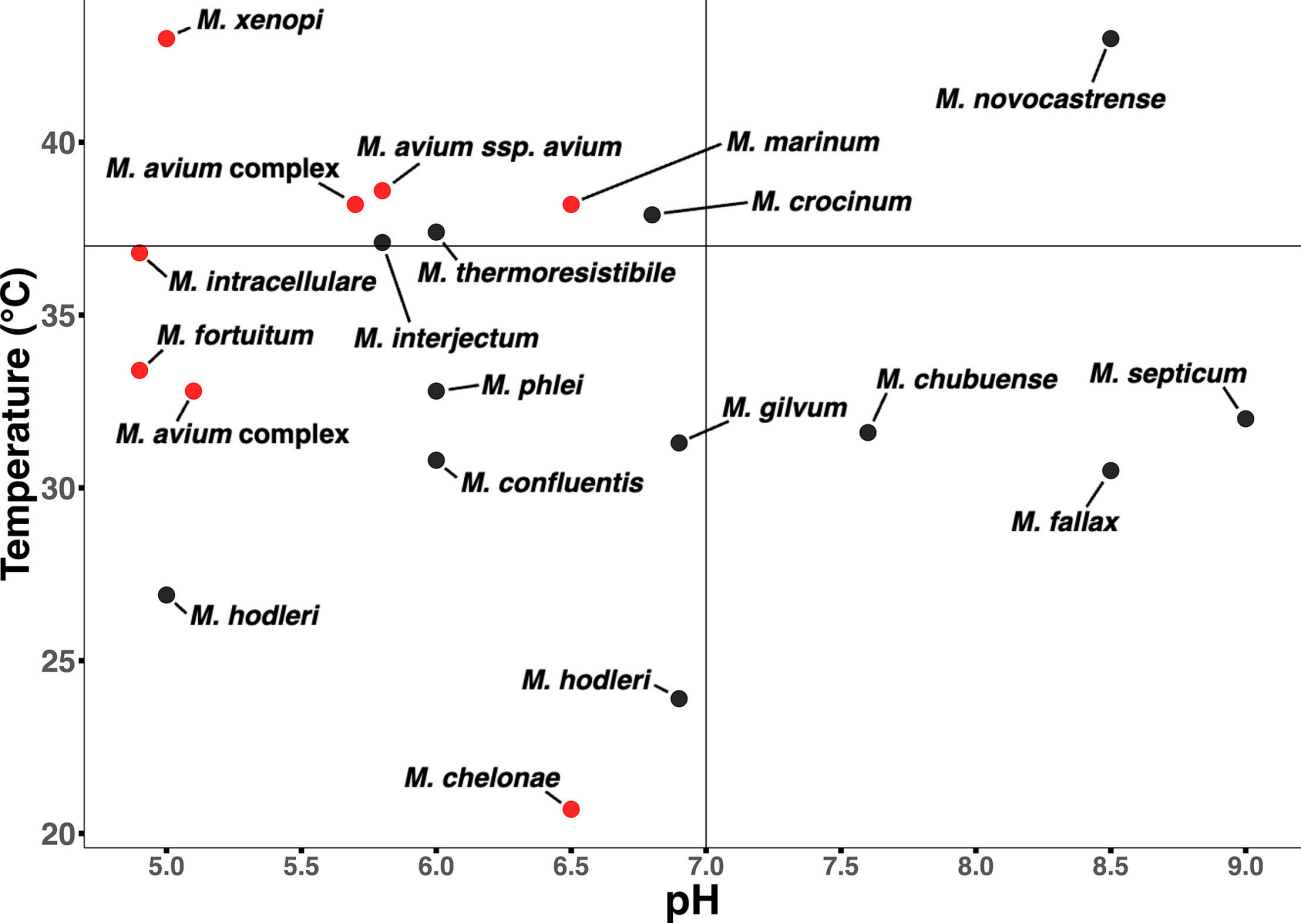
Quadrant scatterplot of the pH and temperature preferences of all 20 isolates, with lines intersecting at pH 7 and 37°C, the standard clinical culturing conditions for most pathogenic nontuberculous mycobacteria (51). Strains known to cause disease in humans are indicated with red dots, while strains that have limited or no known pathogenicity are indicated with black dots. Although *M. chelonae*, a skin pathogen, has a low temperature optimum and near-neutral pH optimum, the optimal range of growth for the isolate extends to include low pH, human skin temperature conditions, making it an effective skin pathogen (see text and Fig. 1 for details on the optimal range of these NTM isolates). For detailed information on the pH and temperature preferences of all 20 strains, see Table S1.

The temperature optima for growth across the 20 NTM strains, as determined from our *in vitro* assays, varied considerably, ranging from 23°C (*M. hodleri* NTM-003) to 43°C (*M. novocastrense* NTM-014). Similarly, the measured pH optima varied from pH 4.9 (*M. intracellulare* NTM-006) to pH 9 (*M. septicum* NTM-040). For a given strain, the optimal growth temperature varied across the pH gradient, and vice versa, meaning temperature influenced the pH optima, and pH influenced the temperature optima. This highlights the importance of considering both environmental parameters together when assessing the optimal growth conditions of a specific taxon (Fig. 2). We also note that the range in optimal growth (calculated as the range in pH and temperature at which growth was 50% of optimum) was variable across strains. Some strains could grow reasonably well across relatively broad ranges in pH and temperature (e.g., *M. septicum* (NTM-040), Fig. S1), while other strains had far more narrow growth preferences (e.g., *M. fallax* (NTM-027) and *M. thermoresistibile* (NTM-033, Fig. S1). Additionally, we found groupings of highly similar strains (for example, the *M. avium* complex) tended to share similar pH and temperature optima (Fig. 3).

### Pathogenic mycobacteria tend to grow optimally at a low pH and high temperature

We focused this experiment on NTM isolates that span a broad range of phylogenetic diversity within the genus, as well as varying degrees of human pathogenicity, to determine if pH and temperature optima varied between the clinically relevant pathogens and those that are rarely, if ever, reported to cause disease in humans. We tested the hypothesis that pathogens should have growth optima at lower pH levels and higher temperatures (closer to human body temperature), as compared with taxa that are generally non-pathogenic to humans. In general, our results supported this hypothesis, as evident from Fig. 3. We found that the NTM taxa most frequently implicated in pulmonary disease (*M. avium* complex and *M. xenopi*) had optimal growth near or above 37°C and in more acidic conditions (pH ~5.5). Although all pathogens were capable of some growth at more neutral pH and at lower temperatures, the optima of these strains all fell around human body temperature (37°C) and more acidic pH values. In contrast, isolates that are generally considered non-pathogenic (e.g., *M. fallax*, *M. gilvum*, *M. hodleri*) were generally incapable, or had severely inhibited growth, below pH 6 and above 35°C (Fig. 3). We found that pH and temperature were significantly associated with clinical significance (*P* < 0.05), as defined by Daley et al. (8). Three of the isolates we tested are considered to primarily cause cutaneous infections (*M. fortuitum*, *M. chelonae, and M. marinum*). Both *M. fortuitum* and *M. chelonae* had growth preferences at lower pHs and temperatures below 37°C (with both growing well near the pH and temperature of skin (around pH 6 and 30°C, respectively). Again, although the growth optima of *M. fortuitum* and *M. chelonae* were below 37°C, the range of these isolates extends into the low pH, high temperature conditions typical of other pathogenic taxa, suggesting that they can cause pulmonary and extrapulmonary disease in humans, as has been documented (73–75). While *M. marinum* is known to primarily cause cutaneous disease, it is important to note that it grows optimally in the range between 38°C to 40°C, although only at more neutral pHs (between pH 6 and pH 7). This could potentially explain why *M. marinum* does not commonly result in pulmonary infections (76–78).

### pH preferences of NTMs predict their distributions in soil

We next assessed whether the measured pH growth preferences of NTM could be used to predict their distributions in soil, as determined via cultivation-independent sequencing of 143 soils from across the USA that ranged in pH from 4 to 9 (see Materials and Methods). As explained in Materials and Methods, we chose to focus exclusively on pH in this analysis because soil pH exhibits minimal temporal variation, but temperature regimes in surface soils can fluctuate seasonally, and mean annual temperature at a site might not relate to the optimal temperature for NTM growth in a given soil (e.g., a NTM that grows optimally at 30°C may still be able to survive effectively at the site even if the mean temperature at that site is far lower).

**Fig 4:**
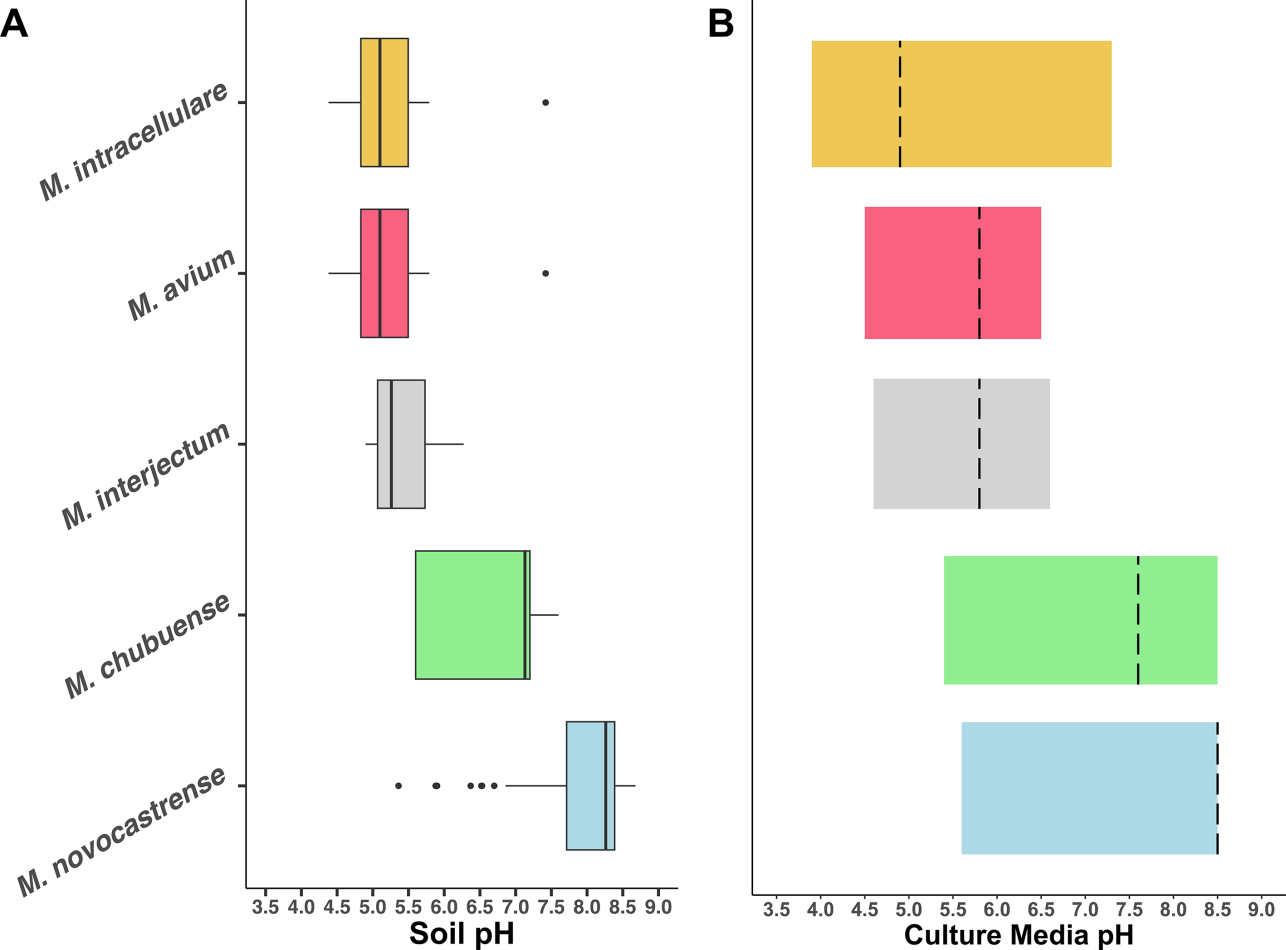
(**A**) The observed distributions of NTM taxa detected in soil, as a function of soil pH (from Walsh et al. [29]) compared with (**B**) the measured in vitro pH growth range and measured growth optima (dashed black line) for the corresponding isolate. The optimal growth value for *M. novocastrense* was determined at the highest pH measured in the experiment.

Five of our assayed isolates were closely related to taxa identified from the cultivation-independent analyses of the soil samples and were well represented across the soil data set. The *M. avium* and *M. intracellulare* isolates (both members of the *M. avium* complex that are of clinical importance) and the *M. interjectum* isolate had measured growth optima at a pH of 5.8, 4.9, and 5.8, respectively. The associated taxa identified in the soil data set were all found to occur primarily in more acidic soils, as expected, highlighting the correspondence between measured pH preferences *in vitro* and observed distribution patterns in soil (Fig. 4). Likewise, the *M. novocastrense* isolate and the *M. chubuense* isolates, which had measured *in vitro* pH optima of 8.5 and 7.6, respectively, matched taxa observed in the cultivation-independent soil data set that were primarily detected in neutral to slightly alkaline soils (Fig. 4). We found a strong, positive correlation (rho = 0.92, *P* < 0.05) between the measured *in vitro* pH optima of the five individual isolates and the mean soil pHs where closely related taxa detected in soil (Fig. 4), highlighting that the expected distributions matched predicted distributions across the soil pH gradient.

## DISCUSSION

Few previous studies have directly measured the pH or temperature preferences of diverse taxa within the genus *Mycobacterium*, with a few exceptions focusing on the growth optima of specific pathogenic and environmental strains (79, 80). Additionally, these previous studies have focused either on pH or temperature independently (27, 81), but not the interaction of the two variables, which are intrinsically interconnected and important when considering potential pathogenicity. We observed substantial variation in both pH and temperature preferences across the 20 NTM isolates investigated (Fig. 1 to 3; Fig. S2). We do not know if this variation in environmental preferences across a given genus is unique, but there are other examples of closely related bacteria that can have distinct pH and temperature preferences, including the genus *Exiguobacterium* (82). However, the observed variation in pH and temperature preferences across the genus *Mycobacterium* is noteworthy, as it could, in part, explain why some NTM species are potentially pathogenic to humans, why the distributions of particular NTM species (including pathogens) can be so variable across non-host environments, including soil (29) and premise plumbing (33), and why some NTM may not be effectively isolated from clinical or environmental samples when using the standard culturing conditions most commonly used to isolate NTM (37°C and pH 7).

As we had hypothesized, almost all the NTM strains that are frequently implicated in pulmonary (or extrapulmonary) disease in humans generally shared similar temperature preferences, growing best at or above human body temperatures (~37°C). *M. avium* subsp*. avium* and *M. xenopi*, with a measured growth optima of 38.6°C and 43°C, respectively (Fig. 3), for instance, are both known to cause pulmonary infections (83, 84). We note that this pattern of pulmonary NTM pathogens having higher temperature optima than other NTM appears consistent regardless of the source of the isolate used in our *in vitro* assays. For example, we included isolates from the *M. avium* complex obtained from different sources, ranging from premise plumbing biofilm (*M. avium* subsp*. avium* [NTM-002] and *M. intracellulare* [NTM-006]) to soil (*M. avium* complex isolates [NTM-036, NTM-038]), yet all shared similar growth preferences (Fig. 3). Other NTM pathogens that frequently cause cutaneous infections (including *M. chelonae*, *M. fortuitum*) shared similarly low pH preferences, but their temperature optima for growth were lower (20°C-33°C) than for the pulmonary pathogens. These differences in temperature optima likely reflect the differences between the human core body temperature (37°C) and human skin temperatures (33°C–37°C) (18) and highlight the importance of temperature in determining tissue tropisms. However, it is important to note that the potential pathogenicity of a strain is also related to the range of conditions at which an NTM can grow near-optimally. For example, *M. chelonae* and *M. fortuitum* primarily cause cutaneous infections, but their broad range in measured temperature preferences highlights their potential capacity to grow in different host tissues. Although *M. chelonae* has a low temperature optimum (~21°C) and a pH optimum ~6.5, it shows near-optimal growth under low pH conditions and at human skin temperatures, making it a potential skin pathogen, as has been noted (85, 86).

Likewise, as we had hypothesized, pathogenic NTM typically had lower pH preferences than the non-pathogenic NTM. The ability to grow in more acidic conditions may allow pathogenic NTM to survive phagocytosis and proliferate within macrophage cells, given that the phagolysosome is typically acidic (~pH 5 [21–23]). However, we note that pH and temperature can interact to shape NTM growth preferences. For example, although *M. marinum* grows well at 30°C under normal skin conditions (30°C–33°C, pH 5–6), we found that it is also capable of growth at higher temperatures (>35°C) under more basic pH conditions (pH > 6). As another example, *M. septicum* grows optimally at ~32°C and under basic conditions (pH > 8), although it grows near optimally under acidic conditions (pH < 6.5) when incubated at cooler temperatures (~23°C). These findings highlight the importance of understanding the interactions between pH and temperature in determining NTM pathogenicity and their potential geographic distributions.

While the discussion above has focused on our analyses of the growth preferences of NTMs that are pathogenic to humans, our results could also help us understand the growth of NTM in protists, including amoeba. Nearly all of the taxa that we identified as having more acidic pH preferences, or showed near-optimal growth under acidic conditions, including *M. chelonae*, *M. fortuitum*, *M. marinum*, *M. septicum*, and the *M. avium* complex (Fig. 2) are capable of survival within amoeboid hosts (87–93). Although temperature optima vary between these NTM strains, all share a similar preference for acidic environments, which could be an important trait allowing for growth inside the acidic vacuoles of amoeboid hosts (87, 91, 94).

Soil is often cited as an important reservoir of NTMs, including pathogenic NTMs. We show that the pH preferences of NTMs measured *in vitro* are associated with the pH range of the soils in which closely related NTM strains were identified from a cultivation-independent survey of 143 soils from across the US (Fig. 4). Although these findings are based on a limited number of NTM taxa (five of the isolates from our study), the correlation between the two measurements effectively highlights that we can use *in vitro* measurements of NTM growth preferences to identify where NTM are most likely to be found in the environment, including habitats which may harbor clinically relevant NTM. Although we focused only on soils here, a clear next step would be to use the measured pH and temperature preferences of NTMs to determine which aquatic systems, another important environmental reservoir of NTM (12, 95, 96), are most likely to harbor pathogenic NTM, information that would likely yield important insights into the epidemiology of NTM disease.

We demonstrate that the growth rates of NTM are highly dependent on specific culturing conditions. This has two important implications. First, the historical designation of NTM species into “slow growers” versus “rapid growers” based on their growth rate at standard culturing conditions (37°C, pH 7) has some limitations since these culturing conditions rarely match the growth optima of many NTM taxa, including some pathogens (Fig. 3). Second, our results show that the “standard” culturing conditions used for the detection of NTMs in clinical samples will likely fail to capture the broad diversity of NTM found in environmental or clinical samples given the observed breadth in pH and temperature growth optima. These findings suggest that a re-evaluation of clinical and diagnostic laboratory practices may be warranted to better capture the potential diversity of NTM and to design better cultivation strategies to isolate and grow pathogenic NTM from either environmental or clinical samples.

While we investigated two important environmental factors (pH and temperature) affecting NTM growth, these are not the only factors that may explain the pathogenicity and geographic distributions of NTM. We recognize that there are other environmental factors that could be relevant to understanding the ecologies of NTM, including oxygen levels and carbon substrate preferences (97–99). Future work quantifying the preferences of NTM species for other environmental factors would enhance our predictive understanding of NTM biogeography and pathogenic potential, including which NTM species can thrive in what types of environments, and why some NTM species can cause infections in humans while others rarely, if ever, cause disease.

## Data Availability

The data sets generated and analyzed for this study can be found in the NCBI BioProject PRJNA1163726. Metadata for these mycobacteria isolates are detailed in Table S1.

## SUPPLEMENTAL MATERIAL

Figure S1 msystems.00211-26-s0001.tiff Additional marker gene phylogenetic trees (*rpoB* and *hsp65*).

Figure S2 msystems.00211-26-s0002.tiff Growth optimum contour plots for 16 additional NTM isolates.

Supplemental Tables msystems.00211-26-s0003.docx Tables S1 and S2.

ASM does not own the copyrights to Supplemental Material that may be linked to, or accessed through, an article. The authors have granted ASM a non-exclusive, world-wide license to publish the Supplemental Material files. Please contact the corresponding author directly for reuse.
