# Supplementary material for: Temperature and pH growth preferences predict the pathogenicity and environmental distributions of nontuberculous mycobacteria
Source: mSystems. 2026 Jul 21;11(8):e00211-26. doi: 10.1128/msystems.00211-26 (PMC13483362; doi:10.1128/msystems.00211-26)
Supplement: Supplementary file 1 [file msystems.00211-26-s0003.docx]

| Isolate ID | Closest Taxonomic Neighbor (Projected Species) | Genome Size | Source Environment | Experimental Timepoint | pH Optimum | Temperature Optimum (℃) | Optimal pH Range (50%) | Optimal Temperature Range (50%) | Model Fit (R-squared) | Pathogenicity | Site of Pathology | Citation (for reported pathogenicity) |
| --- | --- | --- | --- | --- | --- | --- | --- | --- | --- | --- | --- | --- |
| NTM-001 | *M. hodleri* | 6.9MB | Soil | Day 14 | 5 | 26.9 | 4.3 - 6.1 | 14.9 - 34.2 | 0.77 | Not reported | NA |  |
| NTM-002 | *M. avium ssp. avium* | 5.6MB | Municipal biofilm | Day 14 | 5.8 | 38.6 | 4.5 - 6.5 | 31.7 - 43 | 0.88 | Yes | Pulmonary | Griffith et al, 2007, ATS Statement |
| NTM-003 | *M. hodleri* | 6.9MB | Soil | Day 14 | 6.9 | 23.9 | 5.3 - 8.5 | 14.4 - 34.2 | 0.77 | Not reported | NA |  |
| NTM-006 | *M. intracellulare* | 5.5MB | Municipal biofilm | Day 7 | 4.9 | 36.8 | 3.9 - 7.3 | 14.6 - 43 | 0.98 | Yes | Pulmonary | Griffith et al, 2007, ATS Statement |
| NTM-010 | *M. chelonae* | 5.2MB | Municipal biofilm | Day 14 | 6.5 | 20.7 | 3.9 - 8.5 | 13 - 34.9 | 0.82 | Yes | Cutaneous, pulmonary | Griffith et al, 2007, ATS Statement |
| NTM-012 | *M. gilvum* | 5.9MB | Municipal biofilm | Day 14 | 6.9 | 31.3 | 5.9 - 8.5 | 19.3 - 36.5 | 0.62 | Not reported | NA |  |
| NTM-014 | *M. novocastrense* | 6MB | Municipal biofilm | Day 7 | 8.5 | 43 | 5.6 - 8.5 | 37.2 - 43 | 0.62 | Very rare | NA |  |
| NTM-017 | *M. xenopi* | 4.9MB | DSMZ; Type Strain (Adult female toad) | Day 7 | 5 | 43 | 3.9 - 8.1 | 30.6 - 43 | 0.94 | Yes | Pulmonary | Griffith et al, 2007, ATS Statement |
| NTM-024 | *M. confluentis* | 5.9MB | DSMZ; Type strain (Human sputum) | Day 14 | 6 | 30.8 | 5.4 - 9 | 16.5 - 38.5 | 0.89 | Not reported | NA |  |
| NTM-025 | *M. interjectum* | 6MB | DSMZ; Type strain (Cervical lymphadenitis) | Day 14 | 5.8 | 37.1 | 4.6 - 6.6 | 30.5 - 41.3 | 0.94 | Rare | NA |  |
| NTM-027 | *M. fallax* | 4.2MB | DSMZ; Type strain (Water sample) | Day 14 | 8.5 | 30.5 | 6.3 - 8.5 | 24.9 - 34.8 | 0.59 | Not reported | NA |  |
| NTM-028 | *M. chubuense* | 5.9MB | DSMZ; Type strain (Garden soil) | Day 14 | 7.6 | 31.6 | 5.4 - 8.5 | 12.1 - 37 | 0.9 | Not reported | NA |  |
| NTM-032 | *M. marinum* | 6.4MB | ATCC; Type Strain | Day 14 | 6.5 | 38.2 | 4.3 - 7.4 | 29.8 - 41.3 | 0.89 | Yes | Cutaneous | Griffith et al, 2007, ATS Statement |
| NTM-033 | *M. thermoresistibile* | 4.9MB | ATCC; Type Strain | Day 14 | 6 | 37.4 | 5.4 - 7.5 | 23.9 - 42 | 0.94 | Not reported | NA |  |
| NTM-034 | *M. phlei* | 5.4MB | ATCC; Type Strain | Day 14 | 6 | 32.8 | 5.4 - 8.5 | 23 - 40.9 | 0.81 | Not reported | NA |  |
| NTM-036 | *M. avium complex* | 5.9MB | Soil | Day 7 | 5.7 | 38.2 | 3.9 - 9.0 | 23.8 - 41.7 | 0.83 | Yes | Pulmonary | Griffith et al, 2007, ATS Statement |
| NTM-038 | *M. avium complex* | 5.9MB | Soil | Day 14 | 5.1 | 32.8 | 4 - 6.4 | 24.7 - 37.4 | 0.85 | Yes | Pulmonary | Griffith et al, 2007, ATS Statement |
| NTM-040 | *M. septicum* | 7MB | Soil | Day 14 | 9 | 32 | 4.4 - 9.0 | 14.4 - 36.6 | 0.42 | Rare | NA |  |
| NTM-047 | *M. crocinum* | 6.3MB | Soil | Day 14 | 6.8 | 37.9 | 4.5 - 9 | 14.8 - 41.4 | 0.75 | Not reported | NA |  |
| NTM-050 | *M. fortuitum* | 6.3MB | Soil | Day 14 | 4.9 | 33.4 | 3.9 - 8.7 | 11 - 43 | 0.8 | Yes | Cutaneous/Pulmonary | Griffith et al, 2007, ATS Statement |

**Supplementary Table 1: Table with full strain information for each isolate (N = 20).** Taxonomy was assigned to each isolate based on phylogenetic proximity to a representative genome in the Genome Taxonomy Database (gtdb.ecogenomic.org). pH and temperature optimum are listed for each isolate, as well as the optimal growth range (50% from the measured optima). Pathogenicity was assigned based on the ATS guidelines. Strains with reported disease in only a few cases, or those having never been reported to cause disease, have been considered either rarely pathogenic or to have no reported pathogenicity, respectively.

| Isolate ID | Closest Taxonomic Neighbor (Projected Species) | k' | edf | k-index |
| --- | --- | --- | --- | --- |
| NTM-001 | *Mycobacterium hodleri* | 35 | 28.00 | 1.00 |
| NTM-002 | *Mycobacterium avium ssp. avium* | 35 | 33.60 | 0.61 |
| NTM-003 | *Mycobacterium hodleri* | 35 | 28.01 | 0.94 |
| NTM-006 | *Mycobacterium intracellulare* | 35 | 33.85 | 0.45 |
| NTM-010 | *Mycobacterium chelonae* | 35 | 26.24 | 1.02 |
| NTM-012 | *Mycobacterium gilvum* | 35 | 25.85 | 0.92 |
| NTM-014 | *Mycobacterium novocastrense* | 35 | 19.57 | 0.87 |
| NTM-017 | *Mycobacterium xenopi* | 35 | 29.60 | 0.89 |
| NTM-024 | *Mycobacterium confluentis* | 35 | 32.69 | 0.71 |
| NTM-025 | *Mycobacterium interjectum* | 35 | 33.10 | 0.75 |
| NTM-027 | *Mycobacterium fallax* | 35 | 10.50 | 0.97 |
| NTM-028 | *Mycobacterium chubuense* | 35 | 32.30 | 0.87 |
| NTM-032 | *Mycobacterium marinum* | 35 | 34.10 | 0.27 |
| NTM-033 | *Mycobacterium thermoresistibile* | 35 | 30.80 | 1.01 |
| NTM-034 | *Mycobacterium phlei* | 35 | 31.88 | 0.70 |
| NTM-036 | *Mycobacterium avium complex* | 35 | 25.50 | 0.41 |
| NTM-038 | *Mycobacterium avium complex* | 35 | 31.05 | 0.38 |
| NTM-040 | *Mycobacterium septicum* | 35 | 16.70 | 0.92 |
| NTM-047 | *Mycobacterium crocinum* | 35 | 31.92 | 0.81 |
| NTM-050 | *Mycobacterium fortuitum* | 35 | 31.29 | 0.63 |

**Supplementary Table 2: Table with generalized additive model diagnostics for each isolate (N = 20).** Model diagnostics show the output from *gam.check*, run for each model independently. Parameters include k’, effective degrees of freedom (edf), and the k-index.
